# A 1-bit electrically reconfigurable metasurface stirrer (ERMS) for improved reverberation chambers

**DOI:** 10.1038/s41598-025-29555-5

**Published:** 2026-02-23

**Authors:** Yewon Kim, Soojeong Kim, Seungkeun Park, Dongho Kim

**Affiliations:** 1 Antennas and Metasurfaces Lab (AML), Department of AI Convergence Electronics Engineering, Sejong University, Seoul, 05006 Republic of Korea; 2 Electronics and Telecommunications Research Institute, Daejeon, 34129 Republic of Korea

**Keywords:** Electrically reconfigurable metasurface stirrer(ERMS), Reconfigurable metasurface, Field uniformity, Lowest usable frequency(LUF), Reverberation chamber(RC), Varactor diode, Working volume, Engineering, Materials science, Physics

## Abstract

We propose an innovative electrically reconfigurable metasurface stirrer (ERMS) designed to significantly enhance the performance of reverberation chambers (RCs). Our proposed ERMS overcomes the limitations of traditional mechanical stirrers or paddles, such as reduced working volume and high lowest usable frequency (LUF). This is achieved by employing a novel metasurface integrated with varactor diodes, which manipulate the stirrer’s reflection phase without any mechanical movement. The fabricated ERMS consists of 88 metasurface unit cells, which measures 2.4 λ × 3.3 λ × 0.122 λ at 600 MHz. Compared to a conventional mechanical stirrer, the ERMS lowered the LUF by 95 MHz (from 420 MHz to 325 MHz), and the working volume increased by nearly three times (from 0.68 m³ to 1.94 m³), thanks to its superior field-stirring characteristics and low-profile planar design. Measurement results confirm that the ERMS significantly improves the capability of RCs, exhibiting an acceptably low standard deviation of fields. This achievement enables more accurate and efficient testing of antennas and wireless communication devices.

**Supplementary Information:**

The online version contains supplementary material available at 10.1038/s41598-025-29555-5.

## Introduction

It is well known that a reverberation chamber (RC) serves as a versatile measurement facility for wireless communication devices and antennas^1,2^. This facility can simulate various radio wave environments and is particularly used to test many communication devices in complex environments such as urban or indoor areas^3–10^.

Three key parameters influence the performance of an RC: field uniformity, lowest usable frequency (LUF), and working volume. First, field uniformity refers to the degree to which electromagnetic fields are evenly distributed within an RC. It is important because it ensures that measurements are consistent and reliable across the chamber.

Next, the LUF, which is the starting frequency of the usable frequency range, is determined experimentally based on the size of an RC and field uniformity^11,12^. Generally, the total number of modes in an RC is proportional to the size of the RC. Accordingly, to maintain the availability of an RC at low frequencies, the RC should be increased, which is challenging from both practical and economic perspectives.

The third parameter to consider is the working volume, which refers to the available volume that guarantees acceptably low field uniformity, allowing target devices-under-test to be placed. In general, the working volume is defined as a specific region within an RC where the field uniformity, measured by a standard deviation of less than 3 dB, can be achieved. To mitigate boundary effects, this region is typically set to be at least λ/4, or more precisely λ/2, away from any boundary of the chamber^11^. Here, the boundary includes conducting walls, diffusers, stirrers, paddles, and other similar devices. Expanding the working volume of an RC is crucial because it enables the measurement of larger devices^13^.

To achieve a low uniform distribution within an RC, mechanical stirrers are typically employed. For these stirrers to be effective, their size must be comparable to or larger than the wavelength of the lowest operating frequency of the RC^12,14–16^. However, they not only reduce the working volume but also increase the RC size to maintain the same LUF. Furthermore, they complicate an RC, leading to increased installation costs.

To solve these problems, mechanical stirrers combined with passive metasurfaces^17–19^ have also been proposed, which effectively lower the LUF. However, they still require large mechanical stirrers.

For this reason, electrical stirrers have been reported in recent studies^20^, which scramble various modes by changing the reflection phase electrically. Although electric stirrers offer a convenient alternative to mechanical stirrers, further research is still needed to fully understand their capabilities and limitations, such as their narrow frequency range, small working volume, and high power consumption.

**Fig. 1:**
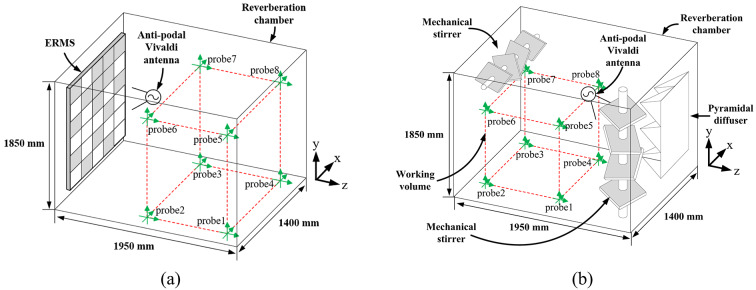
The operation of an RC using (**a**) the proposed planar ERMS, and (**b**) conventional mechanical stirrers and six metallic diffusers.

To overcome these problems, we propose a novel 1-bit electrically reconfigurable metasurface stirrer (ERMS), as illustrated in Fig. 1a. The proposed ERMS meets international standards by achieving a 3 dB field uniformity limit within the frequency band of interest. It features a lowered LUF of 325 MHz, which is 95 MHz lower than that of conventional mechanical stirrers in an RC of the same size. Additionally, its working volume is 1.94 m³, which is nearly three times larger than that of the RC with the mechanical stirrers. This significant improvement is enabled by the ERMS’s superior field-stirring characteristics and its low-profile planar design. Notably, our ERMS requires only a simple 1-bit operation to acquire such remarkable performance. All simulation results were obtained using the CST Studio Suite^21^.

## Design and electrical stirrer

### Operation principle

Field uniformity in an RC is quantified by the standard deviation (σ) of electric (*E*) fields, as indicated by Eq. (1), where ‘*i*’ denotes the specific position of a measurement probe within a test volume^12^.

1 $$\sigma = \sqrt {\frac{{\sum\limits_{{i = 1}}^{8} {\left( {E_{i} - \left\langle E \right\rangle _{8} } \right)^{2} } }}{{8 - 1}}}$$

The ensemble average of measured fields during the rotation of mode stirrers is represented by <·>. Ideally, as σ approaches zero, the resulting field uniformity stabilizes. However, in practice, σ is recommended to be kept lower than 3 dB above 400 MHz^12^. To keep this limitation, conventional RCs have used mechanical stirrers and paddles, static scatterers or diffusers, etc. These components, which must have dimensions comparable to or larger than the wavelength at LUF, inevitably reduce the available working volume of the RC.

**Fig. 2:**
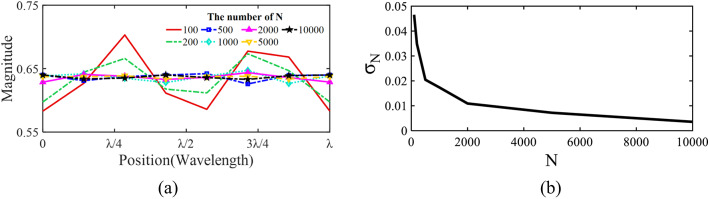
(**a**) The distribution of the averaged magnitude according to the number of random-phased signal samples. (**b**) Average of standard deviations for the number of the signal samples.

The operational principle of the proposed method is conceptually explained in Fig. 2. As more random-phase sinusoidal signals are summed, the magnitude deviation—the difference between the maximum and minimum resultant magnitudes—decreases with an increasing total number (*N*) of signals, which is given in Fig. 2a.

The standard deviation (σ_N_) of Fig. 2a according to *N* is shown in Fig. 2b, which has been calculated using (1) by replacing the number “8” with *N*. As we can find, σ_N_ converges to zero as *N* increases, indicating that a sufficient number of random signals can effectively stir the fields, leading to a significant reduction in standard deviation. In Fig. 2a, it is also important to note that the resulting magnitudes of the mixed fields do not decrease below a specific value as *N* increases, which is crucial for maintaining field strengths above a meaningful level. The proposed ERMS provides random phase offsets to keep fields in an RC as uniform as possible. This is the main idea of our work.

## ERMS design

**Fig. 3:**
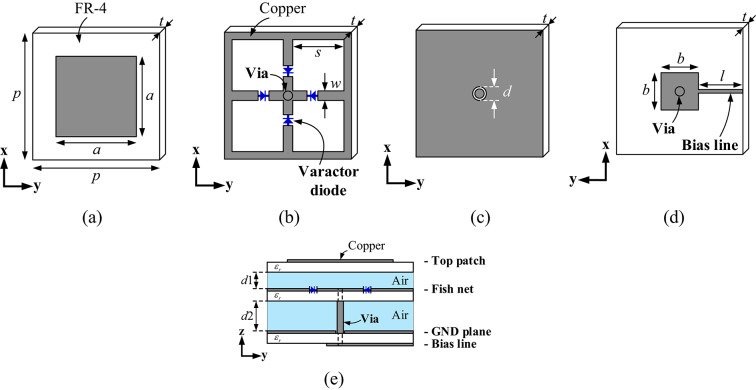
Geometry of the proposed metasurface unit cell with (**a**) a top layer, (**b**) a middle layer, (**c**) the top face of a bottom layer with a ground plane, (**d**) the bottom face of the bottom layer with the DC bias line, and (**e**) the side view of the unit cell. The design parameters are *p* = 150 mm, *t* = 2 mm, *a* = 120 mm, *w* = 2 mm, *s* = 71.5 mm, *m* = 10 mm, *b* = 30 mm, *l*= 60 mm, *d*_1_ = 15 mm, *d*_2_ = 40 mm, and *ε*_r_= 4.3.

The unit cell geometry of the proposed ERMS, consisting of three layers, is shown in Fig. 3. All layers are made from a 2 mm-thick FR-4 laminate with a dielectric constant of 4.3 and a loss tangent of 0.025. The unit cell measures 150 mm × 150 mm × 61 mm, corresponding to 0.3 λ × 0.3 λ × 0.122 λ at 600 MHz, the center frequency of the operating frequency band, which ranges from 350 MHz to 900 MHz.

The top layer, consisting of a square copper patch, is shown in Fig. 3a. The middle layer (Fig. 3b) under the top layer consists of a fishnet structure containing four varactor diodes from Skyworks SMV1405-040LF. To maintain the four varactor diodes in their reverse-bias region, a positive DC bias voltage is applied to the central cross line through the central via, which is connected to the bias line on the bottom layer, as shown in Fig. 3d. Meanwhile, the outer square loop is connected to the ground plane (Fig.3c) using jump wires (not shown), which is crucial for providing exact DC bias voltages to the diodes, thereby stabilizing their operation. The bottom layer comprises a ground plane (Fig. 3c) and a DC bias line (Fig.3d), which is connected to the via of the middle layer (Fig. 3b). In Fig.3c, a narrow circular slit is placed between the via and the ground plane to avoid an electrical short. The side view of the proposed unit cell is also given in Fig. 3e. Given the bilateral symmetry of the unit cell in the *xy* plane, the reflection coefficient is the same for both *x*- and *y*-polarized incident waves.

### Design of reflection phase groups for electrical stirring

**Fig. 4:**
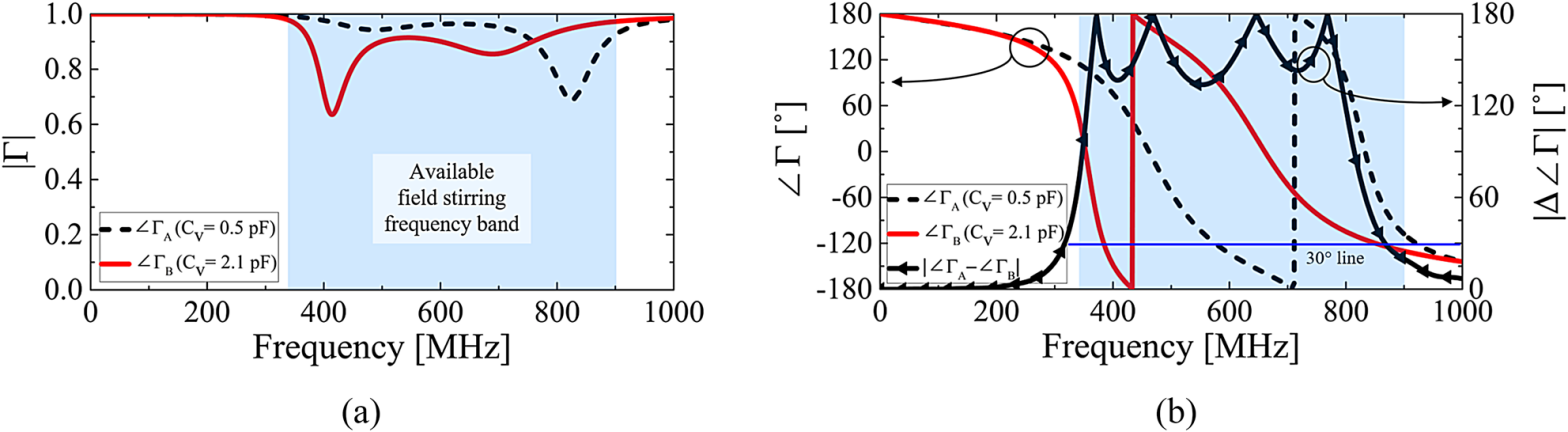
**(a)**Reflection magnitudes for the final varactor capacitances of *C*_v_ = 0.5 pF and 2.1 pF, and **(b)** the reflection phase of the ERMS unit cell and phase difference (∣Δ∠Γ∣) for the two *C*_v_ values.

The proposed ERMS consists of 88 unit cells, with a total of 352 varactors, as each unit cell contains four varactors. The unit cells are classified into two groups, providing different reflection phases Δ∠Γ_A_ and Δ∠Γ_B_, respectively. For simplicity, we will denote Δ∠Γ = Δ∠Γ_A_ - Δ∠Γ_B_. Moreover, we have intentionally designed Δ∠Γ to satisfy 90° ≤ |Δ∠Γ| ≤ 180°, for enhanced field uniformity. In other words, all the cells in the ERMS provide at least a 90° phase difference. The rationale for choosing the upper limit of 180° is clear: two signals with an equal magnitude and a 180° phase difference consistently result in perfect destructive interference at any co-located observation point. However, in RCs, signals arrive at any point via numerous different propagation paths, inherently leading to a wide range of possible phase delays. Therefore, a lower limit of 90° (corresponding to a quarter-wavelength phase delay) is intentionally selected as the midpoint between 0° and 180°. This lower limit can be refined subsequently to achieve the required σ﻿ lower than or equal to 3 dB. Although detailed explanations will follow, experiments have shown that the lower limit of 30° is sufficient for field steering, as demonstrated in Fig. 4b.

At each electrical stirring step, we randomly arranged the cells to mix the fields in an RC effectively. Accordingly, we can maintain σ﻿ as low as possible, as demonstrated in Fig. 2.

### Dual-resonance optimization in the multilayer unit cell

The reflection magnitude and phase of the proposed unit cell for the two varactor capacitances (*C*_v_ = 0.5 pF and 2.1 pF), which were used in this work, are given in Fig. 4. Basically, our unit cell structure provides different reflection phases for different *C*_v_ values, thereby functioning as an artificial magnetic conductor (AMC)^22^. However, in AMCs, there are two inherent problems when attempting to manipulate reflection phase values: a narrow frequency variation range that provides the required phase difference of 180°, and a reduced reflection magnitude around a resonance frequency^23,24^. The narrow frequency range restricts the available stirring frequency range, and the decreased reflection magnitude should be avoided for comprehensive EMC tests in RCs. However, the target frequency band is too wide for the proposed ERMS to effectively stir fields, as it ranges from 350 MHz to 900 MHz, corresponding to approximately a 100% fractional bandwidth (FBW). To solve this problem, we devised a multilayer unit cell geometry, as shown in Fig. 3, which simultaneously maximizes reflection magnitude and field stirring frequency range.

For the solid red line (*C*_v_ = 2.1 pF) in Fig. 4a, there are two resonances at about 400 MHz and 700 MHz, at which the reflection magnitudes fall into two local minima. The resonant frequencies should be as far apart as possible to achieve a wider frequency coverage. Similarly, the two minima must be maximized for the practical usage of RCs. To accomplish these goals simultaneously, we optimized the two resonances. The middle fishnet layer (Fig. 3b) causes the first resonance at 400 MHz, and the top patch layer (Fig. 3a) induces the second resonance at 700 MHz, respectively.

A ground plane (Fig. 3c) is essential for the middle fishnet and the top square patch to function as an AMC. An AMC structure generates two distinct reflected waves: one reflected from the upper structure and the other from the lower ground plane. Ideally, when these two waves become in phase in the desired direction, the reflection phase approaches 0°. However, at this 0° phase point, the reflection magnitude is minimized due to material losses experienced as the two signals simultaneously approach their maxima. Consequently, the spacing parameters, *d*_1_ and *d*_2_, play a crucial role by ensuring that the resonance frequencies (at which the reflection phase is 0°) are adequately separated. Consequently, by adjusting the two air spacings, *d*_1_, and *d*_2_, we can control the frequency separation distance between the resonances, thereby optimizing the phase slope for broader bandwidth utilization, as shown in Fig. 4b.

spacings, *d*_1_, and *d*_2_, we can control the frequency separation distance between the resonances, thereby optimizing the phase slope for broader bandwidth utilization, as shown in Fig. 4b.

Figure 4b shows the final reflection phases obtained from the two varactor capacitances used in this work:*C*_v_ = 0.5 pF and 2.1 pF. The phase difference (|Δ∠Γ|) between them is also provided. The |Δ∠Γ| was designed to be larger than at least 90° within the target frequency band, to guarantee lower σ. It is important to note that only 1-bit bias voltages are sufficient to obtain large |Δ∠Γ| as shown in Fig. 4b.

**Fig. 5:**
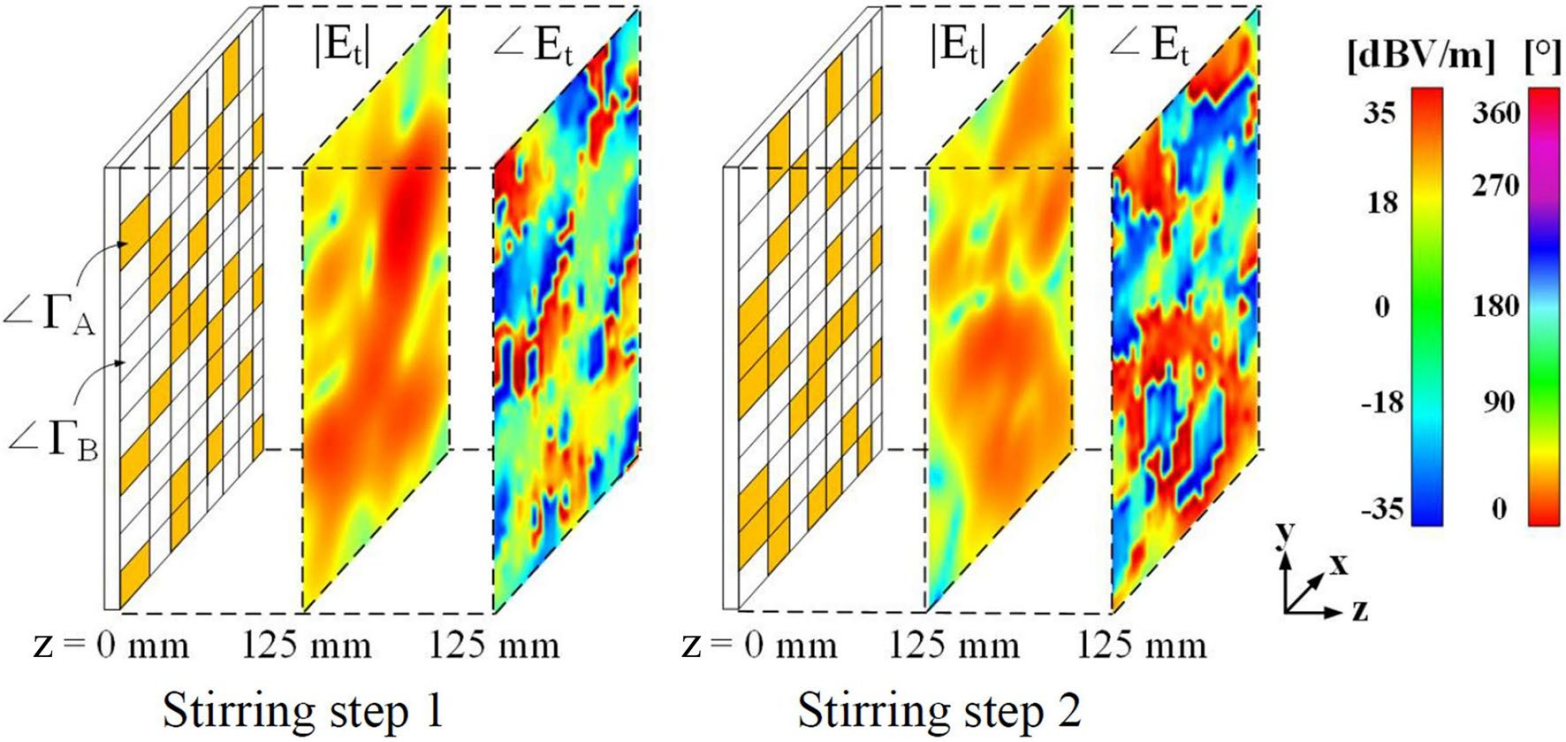
Conceptual diagram of a random arrangement of 8 × 11 unit cells providing different reflection magnitudes and phases. The incident wave propagates perpendicularly to the *xy*-plane in the -*z* direction.

Now, we need to assign the required |Δ∠Γ| to the ERMS, which is conceptually demonstrated in Fig. 5. In the figure, a *y*-polarized plane wave coming from the + *z* direction is used as an incidence, and the corresponding magnitude and phase distribution of total electric fields were simulated. As shown in the figure, unit cells providing ∠Γ_A_ and ∠Γ_B_ were randomly arranged, and at each stirring step, the cells with ∠Γ_A_ and ∠Γ_B_ are placed randomly. As shown in Fig. 5, the two example stirring steps yield completely different distributions, demonstrating high field uniformity with a desirable low standard deviation (σ﻿). In this work, we stirred a total of 24 times for the entire frequency band of interest. An example of DC bias voltages applied to each bias port at each stirring step is shown in Table S1 of the supplementary document.

## Fabrication and measurements

**Fig. 6:**
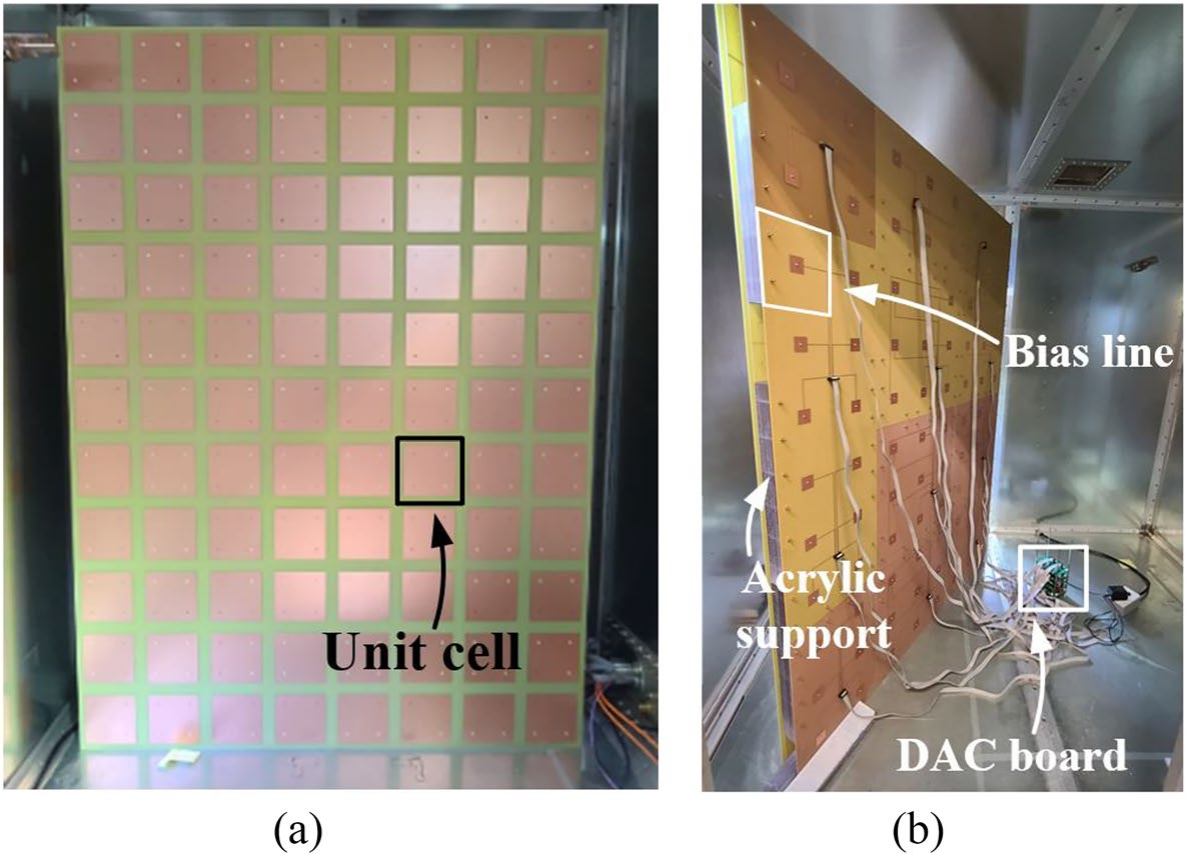
(**a**) The front side and (**b**) the rear side of the fabricated ERMS.

**Fig. 7:**
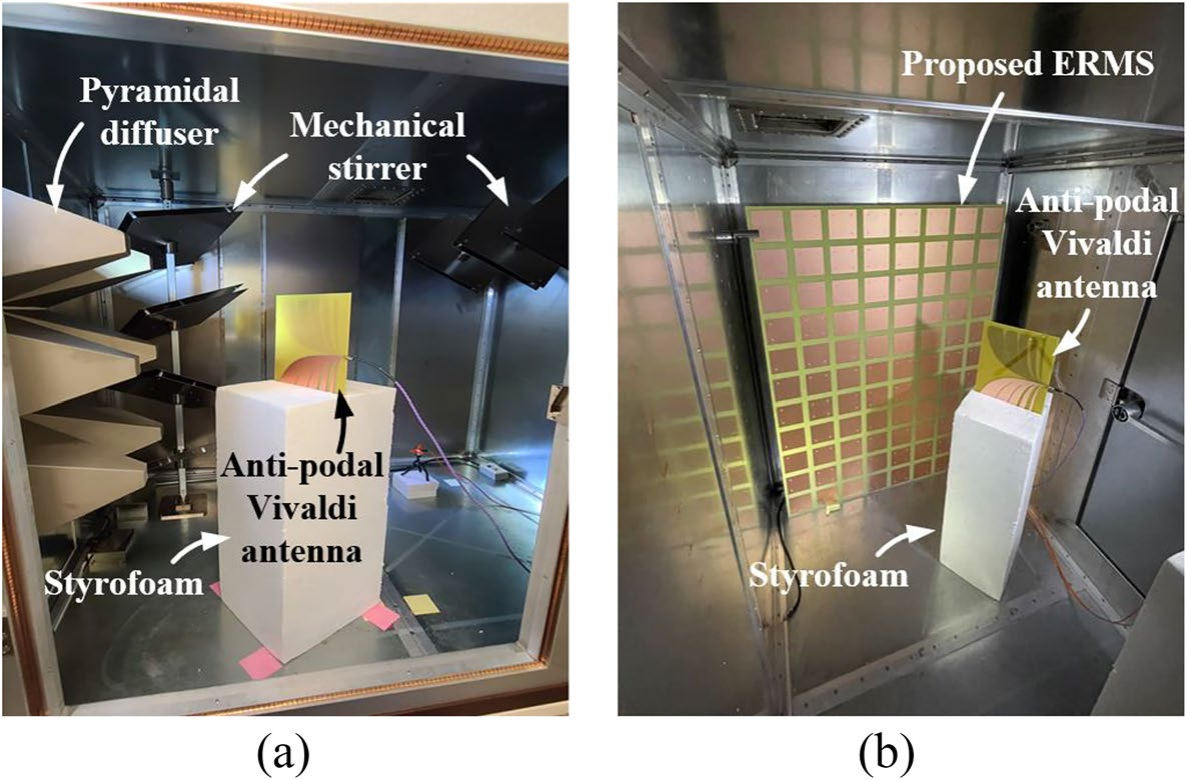
(**a**) The measurement environment with the mechanical mode stirrers and diffusers in the RC, and (**b**) with the ERMS in the same RC with no mechanical stirrers or diffusers.

The fabricated ERMS is shown in Fig. 6. The proposed low-profile planar ERMS consists of 88 unit cells, which measure 1,200 mm × 1,650 mm × 61 mm, corresponding to 2.4 λ × 3.3 λ × 0.122 λ at 600 MHz. Given its large size, we divided the ERMS into twelve boards: six 450 × 450 mm² boards, five 450 × 300 mm² boards, and one 300 × 300 mm² board. Then, we assembled each board using acrylic supports, plastic bolts, and nuts. Figure 6b shows the back side of the ERMS, biasing circuits, and lines. Each unit cell is connected to the DC power supply, which consists of digital-to-analog converters (DAC8718), microcontroller units (STM32F103), and a power supply. The detailed layout of the bias circuit for applying 1-bit DC bias voltages, associated with Figs. 3c &d, is provided in Fig. S3 of the supplementary document. Figure 7a depicts the measurement setup of the conventional RC, which includes horizontal and vertical mode stirrers, three metallic plates, and six pyramidal diffusers. The RC measures 1,400 mm × 1,850 mm × 1,950 mm, and the two horizontal and vertical mechanical stirrers measure 460 mm × 1,850 mm × 460 mm and 1,400 mm × 400 mm × 400 mm, respectively. Each mechanical stirrer rotates with 15° intervals during the measurement, providing 24 stirring steps, which is identical to the total stirring number of the proposed ERMS.The experimental setup using the proposed ERMS is also shown in Fig.7b. Only one ERMS was installed on one wall with no mechanical stirrers or diffusers. A 1-bit control randomly applied two different external DC bias voltages—30 V (*C*_v_ = 0.5 pF) and 0.7 V (*C*_v_= 2.1 pF)—to the two groups of unit cells, providing the required large |Δ∠Γ|^25^.

**Fig. 8:**
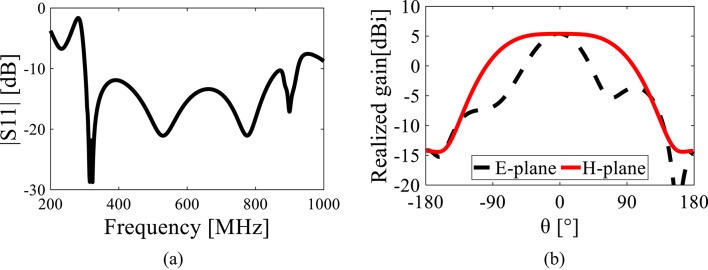
(**a**) Reflection coefficients (S11) and (**b**) radiation patterns (at 600 MHz) of the antipodal Vivaldi antenna used as a signal source.

As with the conventional stirrers, we also used the same number of 24 stirring steps, resulting in different random distributions of the unit cells. We used MATLAB’s random number generation function with a uniform distribution for the stirring.

**Fig. 9:**
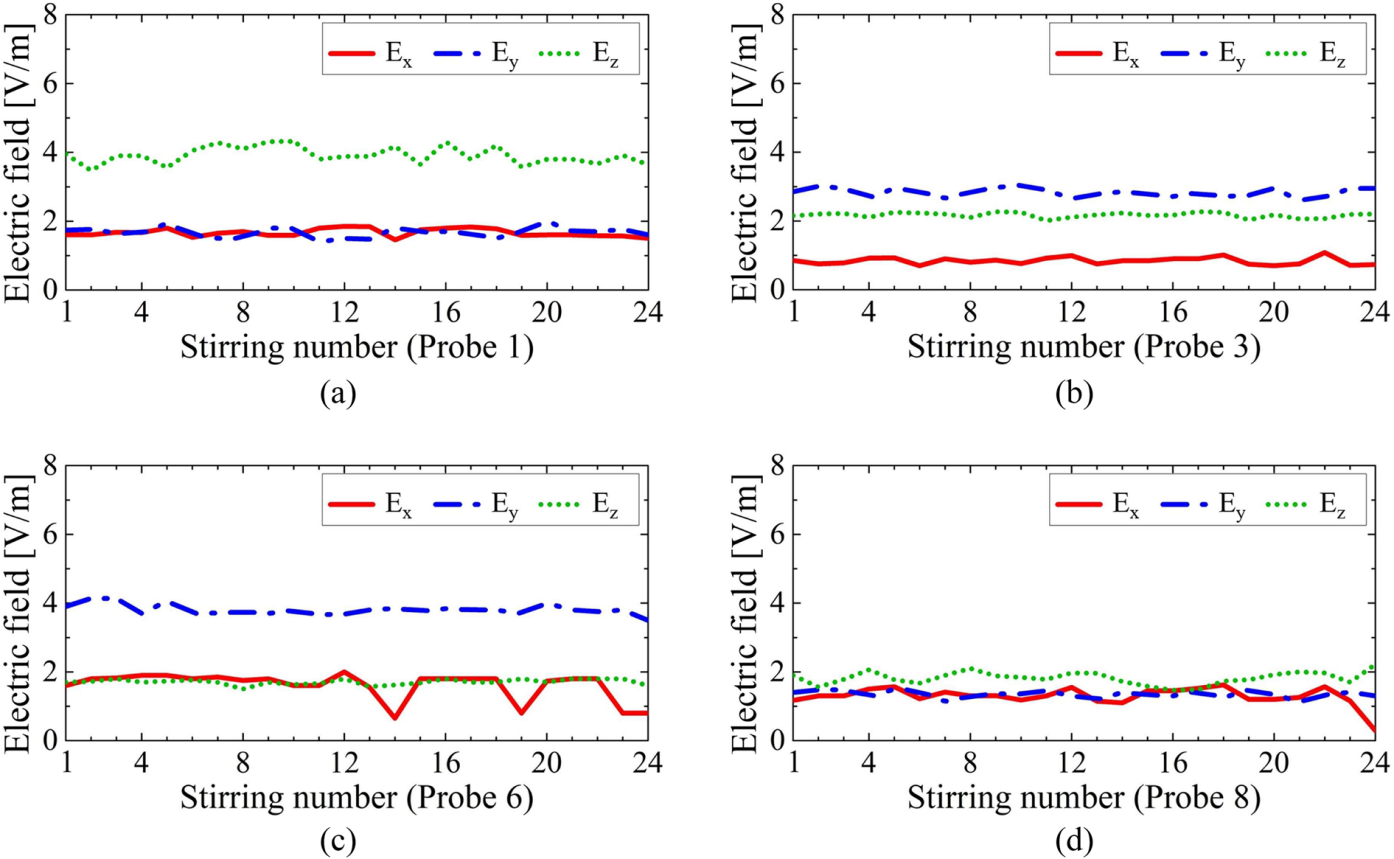
Magnitude of the electric field using the proposed ERMS. All the fields were measured at 200 MHz at the location of (**a**) probe 1, (**b**) probe 3, (**c**) probe 6, and (**d**) probe 8.

To evaluate the performance of the ERMS, we compared the standard deviation measured using either the conventional mechanical stirrers or the ERMS, with only one being used in the same RC at a time. Here, it is worth noting that the ERMS was installed only on one wall with no mechanical stirrers or diffusers, as shown in Fig. 7b.

We used an antipodal Vivaldi antenna as the source antenna, operating in the frequency range of 300 MHz to 930 MHz, as shown in Fig. 8. The precise fabrication geometry of the antenna and its 3D radiation patterns at several key frequencies are both presented in Figs. S1 and S2 of the supplementary document. During the measurements, the antenna was facing each stirrer outside the working volume, as depicted in Fig. 7. An Agilent E4438C signal generator was used as a source signal, and electric fields were measured using an FL7006 broadband probe from Amplifier Research (AR). The probe was positioned at each of the eight vertices of the working volume to measure the *x*, *y*, and *z* components, as indicated in Fig. 1(a)^11^.

**Fig. 10:**
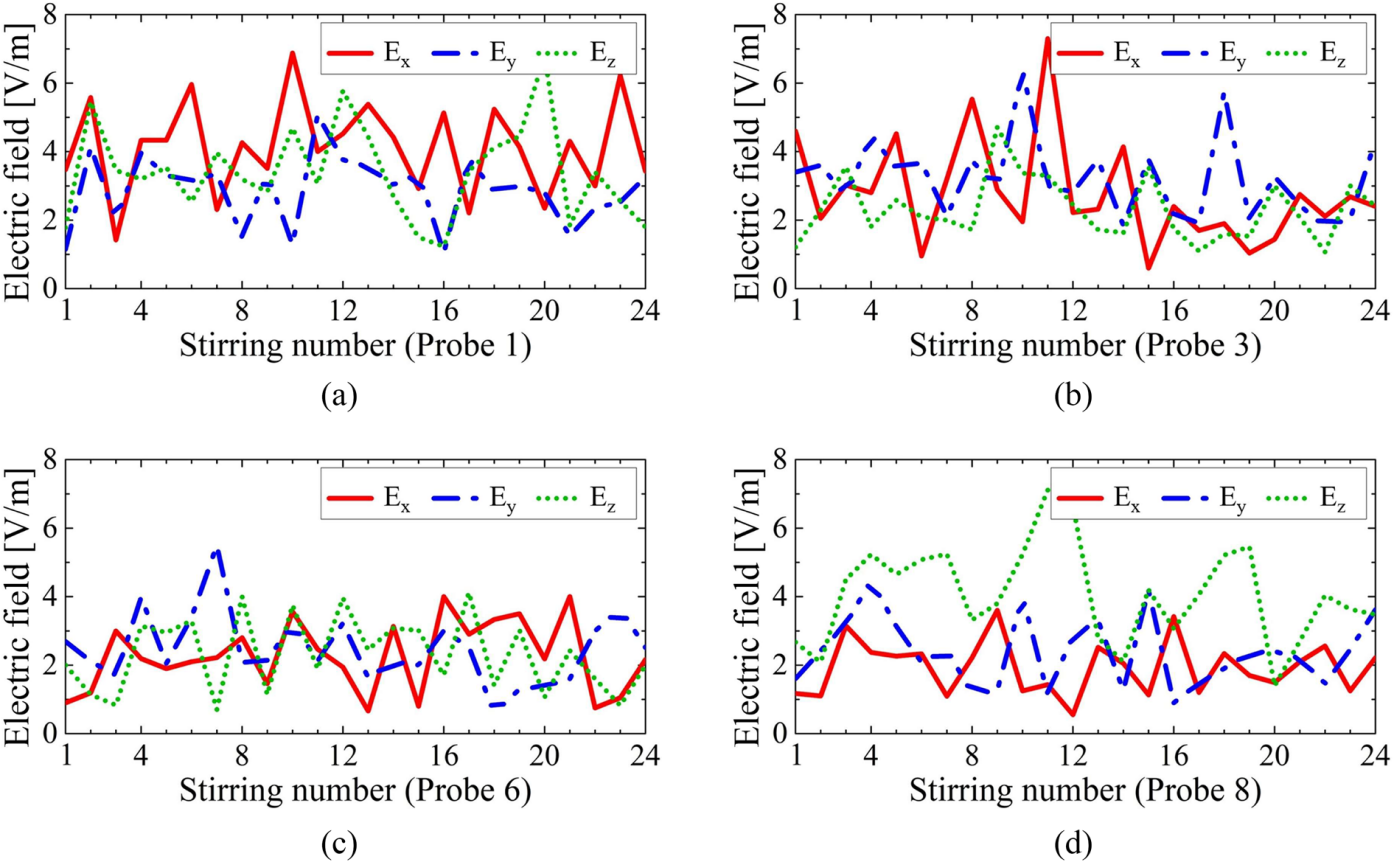
Magnitude of the electric field using the proposed ERMS. All the fields were measured at 600 MHz at the location of (**a**) probe 1, (**b**) probe 3, (**c**) probe 6, and (**d**) probe 8.

Figure 9 presents the magnitude of the electric field measured at 200 MHz, which is outside the target frequency band (350 MHz–900 MHz), using the proposed ERMS with 24 stirring steps. The measurements were conducted at the locations of probes 1, 3, 6, and 8, as shown in Fig. 1a. It can be observed that the magnitude of the electric field remains relatively unchanged below the target frequency band at all locations, regardless of the stirring process. Based on these results, the proposed ERMS appears to be ineffective at properly mixing modes within the RC. This is because the reflection phases of the two groups of unit cells remain relatively unchanged at frequencies outside the target band, even when the applied varactor voltages are varied. We can expect these unstirred field distributions from the unchanging phase difference shown in Fig. 4b.

In contrast, Fig. 10 illustrates the electric field measured at 600 MHz, which falls within the target frequency band, using the proposed ERMS. The measurements were performed at the same locations (1, 3, 6, and 8) and under the identical stirring conditions employed for the results in Fig. 9. Unlike at 200 MHz, the magnitudes of the electric fields exhibit significant variations with each stirring operation. This observation confirms that the proposed unit cell provides distinct reflection phases depending on the applied voltage, thereby effectively mixing the modes inside the RC as intended by the operational principle.

**Fig. 11:**
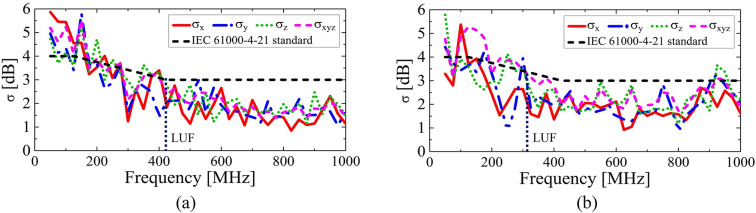
Standard deviation measurement results using (**a**) two conventional mechanical mode stirrers and (**b**) the proposed ERMS.

**Table 1:** Performance comparison of the metasurface mode stirrers.

**Reference**	**Stirring method**	**Fractional bandwidth [%]**	**Operation frequency band [MHz]**	**Advantages**
^17^	Mechanically rotating a passive MS1	86	800–2000	Extended working volume
^18^	Mechanical mode stirrer with passive MS	Upper limit not specified	153–953	Lower LUF
^19^	Mechanical mode stirrer with passive MS	Upper limit not specified	100–200	Lower LUF
^20^	Electrically reconfigurable MS	8	5000 −5400	Extended working volume without mechanical operation
This work	Electrically reconfigurable MS based on varactors	96	325–925	Lower LUF and extended working volume without mechanical operation

The variation in the electric fields with each stirring operation in Fig. 10 effectively decreased the standard deviation of the electric fields inside the RC, as demonstrated in Fig. 2. The measured standard deviation (σ) using the conventional mechanical and the proposed electrical mode stirrers are compared in Fig. 11. Here, we need to focus on two critical performance indicators: the LUF and the working volume. First, let us examine the LUF. The measured LUF with the proposed ERMS is about 325 MHz, which is 95 MHz lower than that with the mechanical stirrer.

Next, let us compare the working volume. The working volume achieved with the EMRS is 1.94 m³ (955 mm × 1,405 mm × 1,444 mm), which is approximately 285% of that with conventional stirrers, which measures 0.68 m³ (955 mm × 839 mm × 854 mm).

Here, we also need to focus on the frequency range from 325 MHz to 925 MHz, where the standard deviation is below 3 dB. This frequency band is closely related to the reflection phase difference (|Δ∠Γ|) shown in Fig. 4b. Although we initially designed |Δ∠Γ| to be greater than at least 90°, it has been proven that 30° is sufficient to achieve an acceptably low σ﻿ as shown in Fig. 4b (refer to the blue straight line).

Lastly, the recent achievements of related studies are summarized in Table I for comparison. As seen in the table, our ERMS operates solely through electrical control without the use of conventional mode stirrers, demonstrating excellent performance in lowering the LUF and extending the working volume over a broad frequency range.

## Conclusions

We propose a simple and effective electrical stirrer that can completely replace conventional mechanical stirrers while meeting international standards. Our stirrer can not only decrease the LUF but also increase the working volume by approximately three times, allowing us to reduce the overall RC size while maintaining the same LUF or working volume.

Although the operational bandwidth of the proposed ERMS spans from 325 MHz to 925 MHz, corresponding to approximately 96% fractional bandwidth, it is possible to widen the bandwidth by installing additional ERMSs that cover higher frequency regions. For example, to cover the frequency range up to 7 GHz, we would need two additional ERMSs with the same fractional bandwidth of 95%, one for the 925 MHz to 2,600 MHz range and another for the 2,600 MHz to 7,300 MHz range. The unit cell size required for AMC operation is proportional to the corresponding wavelength. Therefore, higher operating frequencies necessitate smaller cells, which allows for their insertion between the proposed unit cells. However, operating in higher frequency regimes requires special attention to minimizing both conductor and dielectric losses. Therefore, maintaining adequate reflection performance and field uniformity entails the selection of appropriate materials and the optimization of their structure.

In these regards, the proposed electrical stirrer is cost-effective, easily manufactured, and scalable to higher frequency regions, while simultaneously offering fast operation. Furthermore, the ERMS enhances RC performance, expanding the usable low-end frequency (LUF) and increasing the working volume. Consequently, the proposed ERMS can serve as an excellent alternative to conventional mechanical stirrers, facilitating more versatile and straightforward wireless communication and antenna tests.

## Supplementary Information

Below is the link to the electronic supplementary material.

Supplementary Material 1

## Data Availability

All data generated or analysed during this study are included in this published article. In addition, detailed information about the antenna used in the measurements, the layout of the DC bias voltage supply circuit, and examples of the applied DC bias voltages are provided in the supplementary document.

## References

[1] Yousaf, J. et al. Characterization of reverberation chamber - A comprehensive review. *IEEE Access.* **8**, 226591–226608 (2020).

[2] Serra, R. et al. Reverberation chambers a La carte: an overview of the different mode-stirring techniques. *IEEE Electromagn. Compat. Mag*. **6** (1), 63–78 (2017).

[3] Holloway, C. L. et al. On the use of reverberation chambers to simulate a Rician radio environment for the testing of wireless devices. *IEEE Trans. Antennas Propag.* **54** (11), 3167–3177 (2006).

[4] Wang, Z., Ren, Y., Zhang, Y., Pan, C. & Zhang, X. A novel mode-stirred reverberation chamber design for 5G millimeter wave bands. *IEEE Access.* **9**, 38826–38832 (2021).

[5] Holloway, C. L. et al. Reverberation chamber techniques for determining the radiation and total efficiency of antennas. *IEEE Trans. Antennas Propag.* **60** (4), 1758–1770 (2012).

[6] Xu, Q. et al. A modified two-antenna method to measure the radiation efficiency of antennas in a reverberation chamber. *IEEE Antennas Wirel. Propag. Lett.* **15**, 336–339 (2016).

[7] Xue, W. et al. Frequency stirring effects on reference antenna efficiency measurements in reverberation chambers. *IEEE Trans. Electromagn. Compat.* **66** (5), 1692–1695 (2024).

[8] Mahfouz, M. Z., Vogt-Ardatjew, R., Kokkeler, A. B. J. & Glazunov, A. A. Measurement and Estimation methodology for EMC and OTA testing in the VIRC. *IEEE Trans. Electromagn. Compat.* **65** (1), 3–16 (2023).

[9] Jeon, S. et al. Distances between rats in reverberation chambers used for large-scale experiments. *J. Electromagn. Eng. Sci.* **21**, 148–152 (2021).

[10] Lee, H., Kang, T. W., Choi, J., Hong, Y. P. & Lee, W. Shielding effectiveness of a PC case using Three-Axis Electro-optic sensors. *J. Electromagn. Eng. Sci.* **23** (1), 1–9 (2023).

[11] Xu, Q. & Huang, Y. *Anechoic and Reverberation Chambers: Theory, Design, and Measurements* (Wiley-IEEE, 2019).

[12] Electromagnetic *Compatibility (EMC), Part 4–21: Testing and Measurement Techniques – Reverberation Chamber Test Methods, IEC 61000-4-21* (Int. Electrotechnical Commission, 2011).

[13] Coates, A. & Duffy, A. P. Maximum working volume and minimum working frequency tradeoff in a reverberation chamber. *IEEE Trans. Electromagn. Compat.* **49** (3), 719–722 (2007).

[14] Clegg, J., Marvin, A. C., Dawson, J. F. & Porter, S. J. Optimization of stirrer designs in a reverberation chamber. *IEEE Trans. Electromagn. Compat.* **47** (4), 824–832 (2005).

[15] Zhou, Z. et al. Performance evaluation of oscillating wall stirrer in reverberation chamber using correlation matrix method and modes within Q-bandwidth. *IEEE Trans. Electromagn. Compat.* **62** (6), 2669–2678 (2020).

[16] Chen, K., Xu, Q., Shen, X. & Ren, C. The effect of zigzag boundaries on the reverberation chamber performance. *IEEE Access.* **9**, 145471–145476 (2021).

[17] Sun, H. et al. Metasurfaced reverberation chamber. *Sci. Rep.* **8** (1), 1–10 (2018). 29371675 10.1038/s41598-018-20066-0 PMC5785530

[18] Wanderlinder, L. F., Lemaire, D., Coccato, I. & Seetharamdoo, D. Practical implementation of metamaterials in a reverberation chamber to reduce the LUF. *IEEE 5th Int. Symp. Electromagn. Compat*Beijing, China,. (2017).

[19] Sun, H. et al. Enhancing the number of modes in metasurfaces reverberation chambers for field uniformity improvement. *Sensors* **18** (10), 1–10 (2018). 10.3390/s18103301 PMC6210994 30275373

[20] Gros, J. B., Lerosey, G., Mortessagne, F., Kuhl, U. & Legrand, O. Uncorrelated configurations and field uniformity in reverberation chambers stirred by reconfigurable metasurfaces. *Appl. Phys. Lett.* **118** (14), 144101 (2021).

[21] CST Microwave Studio. (2025). https://www.cst.com/

[22] Dewan, R. et al. Artificial magnetic conductor for various antenna applications: an overview. *Int. J. RF Microw. Comput. -Aided Eng.* **27** (6), e20993 (2017).

[23] Kim-Thi, P., Nguyen, D. T. & Nguyen, T. T. L. A compact and flexible monopole antenna with controllable bandwidth. *J. Electromagn. Eng. Sci.* **24** (5), 443–450 (2024).

[24] Sievenpiper, D., Zhang, L., Broas, R. F. J., Alexopolous, N. G. & Yablonovitch, E. High-impedance electromagnetic surfaces with a forbidden frequency band. *IEEE Trans. Microw. Theory Techn*. **47** (11), 2059–2074 (1999).

[25] Skyworks Solutions Inc. Skyworks Data Sheet. (2018). http://www.skyworksinc.com/

