# Supplementary material for: A 1-bit electrically reconfigurable metasurface stirrer (ERMS) for improved reverberation chambers
Source: Sci Rep. 2026 Feb 23;16:9584. doi: 10.1038/s41598-025-29555-5 (PMC13009375; doi:10.1038/s41598-025-29555-5)
Supplement: Supplementary file 1 — Supplementary Material 1 [file 41598_2025_29555_MOESM1_ESM.docx]

Supplementary Document

A 1-Bit Electrically Reconfigurable Metasurface Stirrer (ERMS) for Improved Reverberation Chambers

**Design and radiation behaviors of the source antenna**

The detailed shape of the wideband antipodal Vivaldi antenna used for signal feeding is given in Fig. S1, which has eight slots loaded for wideband operation. The antenna is printed on a 3.2 mm thick FR4 substrate, which measures 400 mm × 270 mm. Because it is printed on both sides of the substrate, it looks perfectly symmetric as shown in the figure.

The radiation patterns at some critical frequencies simulated using CST Studio Suite are shown in Fig. S2. At 100 MHz, the wavelength is much longer than the antenna size, which is why the radiation pattern appears nearly omnidirectional. From 300 MHz and above, the main beam begins to focus in the boresight direction (left-hand side of the figure) and becomes sharper as the frequency increases, resulting in a higher antenna gain.


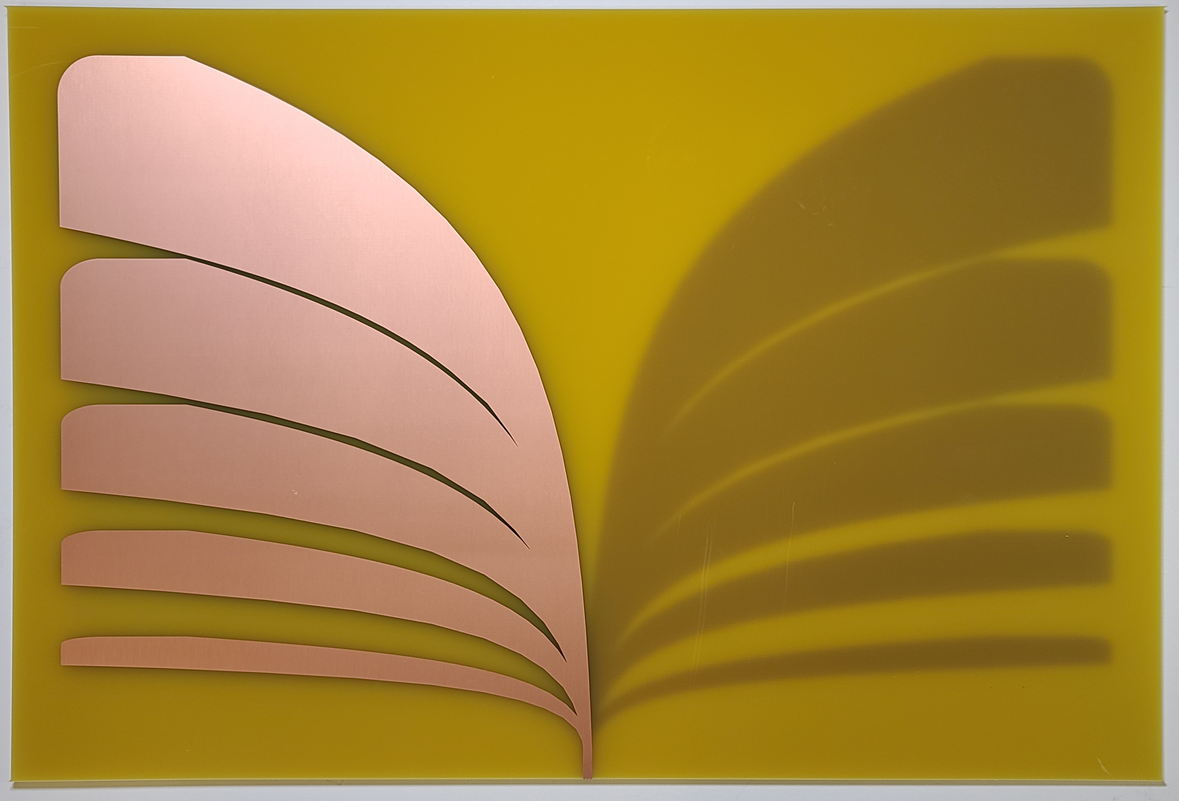

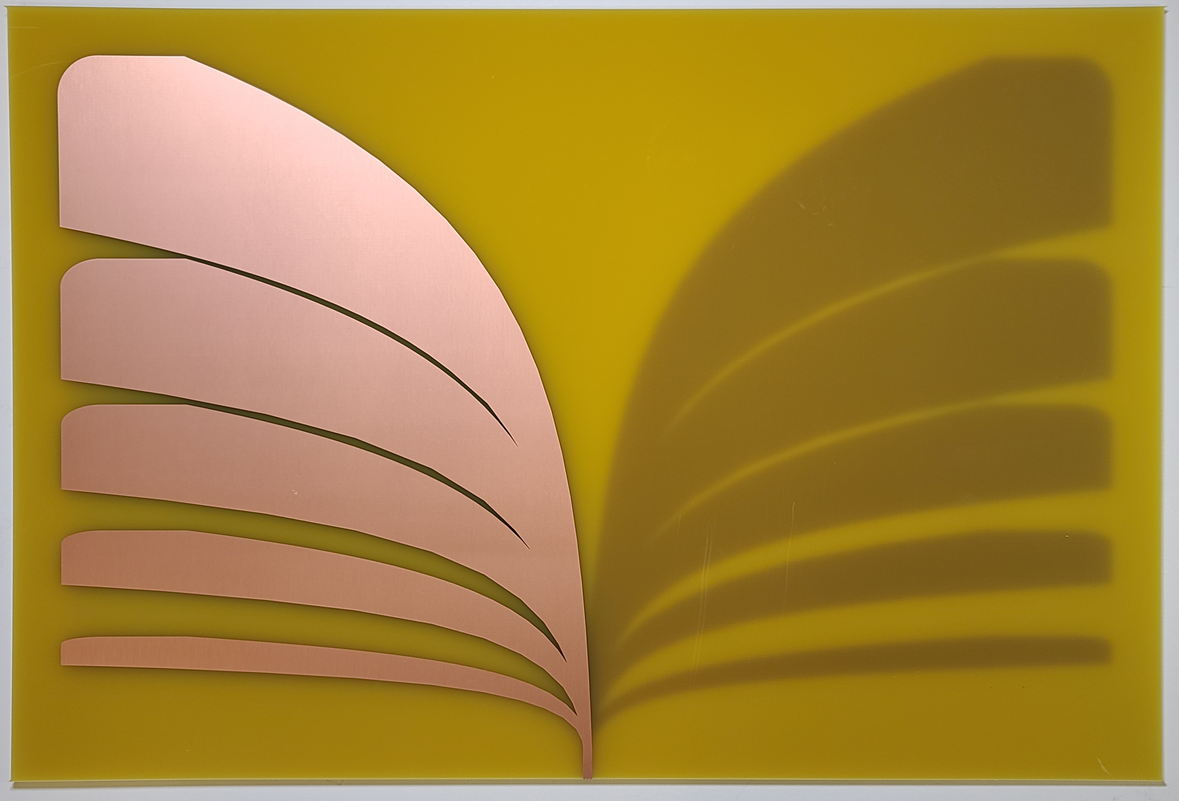


(a) (b)

**Figure S1.** A source-feeding antipodal Vivaldi antenna fabricated on FR4. (a) Front-side view and (b) back-side view.

Enlarging the antenna could achieve a more directional main beam even at 300 MHz; however, this would result in an excessively large antenna size. Such a large antenna would have a negative impact on experiments within the relatively confined space of the reverberation chamber. Therefore, we opted for the current antenna dimensions.

**Layout of the DC bias voltage-supply circuit**


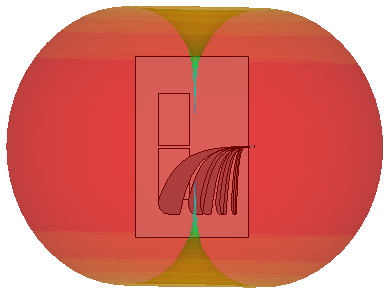

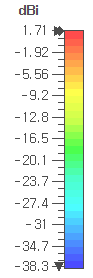

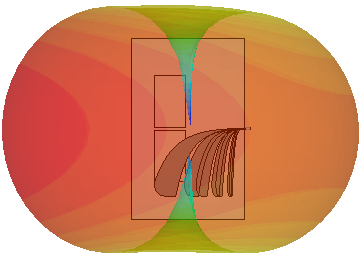

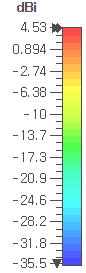


(a) (b)


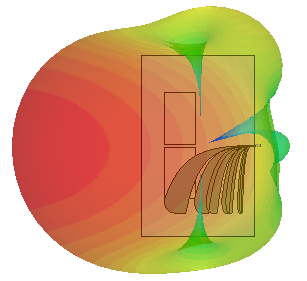

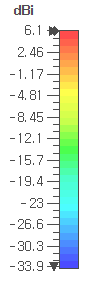

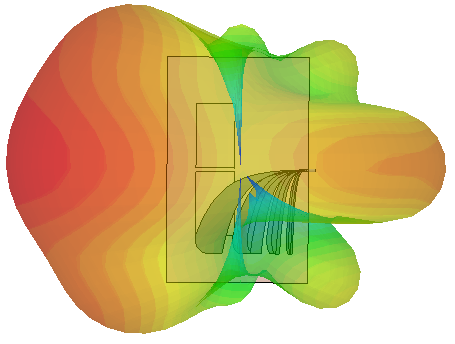

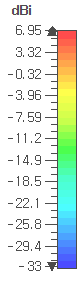


(c) (d)

**Figure S2.** The 3D radiation pattern (realized gain) of the antipodal Vivaldi antenna at (a)100 MHz, (b) 300 MHz, (c) 600 MHz, and (d) 900 MHz.

A part of the DC bias voltage-supply circuit is presented in more detail in Fig. S3. Figures S3(a) and S3(b) depict the circuits corresponding to Fig. 3(c) and 3(d) of the manuscript, respectively. Figure S3(c) shows the cross-sectional view of the DC bias voltage-supply circuit.

As noted in the manuscript, the proposed electrically reconfigurable metasurface stirrer (ERMS) is composed of 88 unit cells. Since each unit cell contains four varactors, the total number of varactors used in the ERMS is 352. To provide the appropriate positive bias voltages to these varactors, we employed the DC bias circuits shown in Fig. S3.

The Digital-to-Analog Converter (DAC) board supplies 1-bit DC voltages to the bias circuit via box header connectors and ribbon cables. The circuit in Figs. S3(a) and S3(b) features nine DC biasing lines that provide two distinct DC voltage levels: 0.7 V (for *C_v_* = 2.1 pF) and 30 V (for *C_v_* = 0.5 pF). For clarity, a few representative bias voltage configurations are summarized in Table S1 as an example.

(a) (b)

(c)

**Figure S3.** Layout of the DC bias voltage-supply circuit. (a) Top-side view showing the traces on the bottom layer, (b) back-side view of the bottom layer, and (c) cross-sectional view.

**Table SI.** Example of DC bias voltages applied to each bias port at each stirring step.

| Bias port  Stirring step | ① | ② | ③ | ④ | ⑤ | ⑥ | ⑦ | ⑧ | ⑨ |
| --- | --- | --- | --- | --- | --- | --- | --- | --- | --- |
| Step 1 | 0.7 | 30 | 30 | 0.7 | 30 | 0.7 | 0.7 | 30 | 0.7 |
| Step 2 | 30 | 0.7 | 0.7 | 30 | 0.7 | 0.7 | 30 | 0.7 | 30 |
| Step 3 | 0.7 | 0.7 | 30 | 0.7 | 30 | 30 | 0.7 | 30 | 0.7 |
| ∙∙∙ |  |  |  |  |  |  |  |  |  |
| Step 24 | 30 | 0.7 | 30 | 0.7 | 30 | 0.7 | 30 | 30 | 0.7 |
